# Electronic Health Physical Activity Behavior Change Intervention to Self-Manage Cardiovascular Disease: Qualitative Exploration of Patient and Health Professional Requirements

**DOI:** 10.2196/jmir.9181

**Published:** 2018-05-08

**Authors:** Deirdre MJ Walsh, Kieran Moran, Véronique Cornelissen, Roselien Buys, Nils Cornelis, Catherine Woods

**Affiliations:** ^1^ Insight Centre for Data Analytics Dublin City University Dublin Ireland; ^2^ School of Health and Human Performance Dublin City University Dublin Ireland; ^3^ MedEx Wellness School of Health and Human Performance Dublin City Unviersity Dublin Ireland; ^4^ Department of Rehabilitation Sciences KU Leuven Leuven Belgium; ^5^ Department of Cardiovascular Sciences KU Leuven Leuven Belgium; ^6^ Department of Physical Education and Sport Sciences Faculty of Education and Health Sciences University of Limerick Limerick Ireland

**Keywords:** telemedicine, exercise, cardiovascular diseases, rehabilitation

## Abstract

**Background:**

Cardiovascular diseases are a leading cause of premature death worldwide. International guidelines recommend routine delivery of all phases of cardiac rehabilitation. Uptake of traditional cardiac rehabilitation remains suboptimal, as attendance at formal hospital-based cardiac rehabilitation programs is low, with community-based cardiac rehabilitation rates and individual long-term exercise maintenance even lower. Home-based cardiac rehabilitation programs have been shown to be equally effective in clinical and health-related quality of life outcomes and yet are not readily available.

**Objective:**

Given the potential that home-based cardiac rehabilitation programs have, it is important to explore how to appropriately design any such intervention in conjunction with key stakeholders. The aim of this study was to engage with individuals with cardiovascular disease and other professionals within the health ecosystem to (1) understand the personal, social, and physical factors that inhibit or promote their capacity to engage with physical activity and (2) explore their technology competencies, needs, and wants in relation to an eHealth intervention.

**Methods:**

Fifty-four semistructured interviews were conducted across two countries. Interviews were audiotaped, transcribed verbatim, and analyzed using thematic analysis. Barriers to the implementation of PATHway were also explored specifically in relation to physical capability and safety as well as technology readiness and further mapped onto the COM-B model for future intervention design.

**Results:**

Key recommendations included collection of patient data and use of measurements, harnessing hospital based social connections, and advice to utilize a patient-centered approach with personalization and tailoring to facilitate optimal engagement.

**Conclusions:**

In summary, a multifaceted, personalizable intervention with an inclusively designed interface was deemed desirable for use among cardiovascular disease patients both by end users and key stakeholders. In-depth understanding of core needs of the population can aid intervention development and acceptability.

## Introduction

### Background

Cardiovascular diseases (CVDs) are a leading cause of early death and disability within Europe and an economic burden worldwide [1]. Importantly, from a behavioral science perspective, approximately 80% of cases are precipitated by lack of self-management of key modifiable risk factors, including physical activity (PA), smoking, diet, alcohol consumption, stress management, and medication adherence [2]. Cardiac rehabilitation (CR) is an essential part of the gold standard management of CVD [3,4] and typically involves risk factor education, supervised exercise training, and psychological support. However, even though CR improves mortality and morbidity rates, uptake of CR remains suboptimal. There are several reasons for the low adherence rates, including travel distance, low self-efficacy, perceived body image, and lack of time [5]. Interestingly, home-based CR programs have been shown to be equally effective in clinical and health-related quality of life outcomes and overcome many of the stakeholder identified barriers to CR participation [6]; however, few CR programs offer a remote solution [7]. This gap in health care provision highlights the need to focus on the needs of individuals living with chronic illness and contemporary CVD self-management.

### New Approaches to Self-Manage Cardiovascular Disease

As current CR delivery approaches do not suit everyone, new innovative ways are needed to match patient preferences and needs to improve uptake and completion of CR. PATHway (Physical Activity Towards Health way) aims to be such an innovative approach. PATHway proposes an Internet-enabled, sensor-based home exercise platform that allows remote participation in CR exercise programs at any time, either by oneself or by a small number of patients, from the comfort of their own living room. The home-based PATHway system will provide an individualized exercise prescription and program leveraging existing technology to facilitate participants to better self-manage their CVD. The proposed technology at this current development phase includes a portable personal computer, including PATHway software complimented by wearable sensors (eg, Microsoft Band 2 heart rate monitor to tailor the PATHway experience.) These sensors will facilitate the participant to engage in tailored exercise classes and games led by an avatar instructor who demonstrates each exercise to be conducted throughout the exercise session.

Saner highlighted how there are many obstacles to implementation, including how development of electronic health (eHealth) interventions is skewed toward technology development rather than user needs and expectations [8]. Therefore, it is important that qualitative development work be recognized as an integral aspect of creating a context specific, fit-for-purpose, user-centered intervention.

It has been identified that incremental stepped approaches to developing and evaluating behavior change interventions are most appropriate as per the Medical Research Council and Behavior Change Wheel frameworks [9,10]. A key tenet of both frameworks is to identify patients’ key personal, social, and physical factors that inhibit or promote their capacity to engage in PA and also to identify their experience, needs, and wants from a technology-based intervention.

Furthermore, to ensure successful implementation of an eHealth behavioral change intervention to self-manage CVD, it is necessary to ascertain the views of key stakeholders from the health care ecosystem and across Europe. It is important to include stakeholders from across the spectrum (as captured by the Social Ecological Model, SEM; [11]), eg, from cardiologists to health policy makers. The SEM incorporates a wide range of individuals involved at various points of the CVD illness journey through its various levels (eg, individual, interpersonal, organizational, community, and policy). This includes those who refer patients to existing CR services and those who deliver those services. Interestingly, this study also seeks to explore the context of the implementation of the PATHway intervention, taking into account the potentially differing health care systems involved in the two sites (ie, Ireland and Belgium). These sites were selected as part of a consortium from European Union’s Horizon 2020 Framework Programme for Research and Innovation Action under Grant Agreement no. 643491.

In an Irish context, CR services have been in development since the 1970’s. CR services in Ireland grew at a fast pace following the implementation of the Building Healthier Hearts program [12]. Both health care systems aim to operate within a multidisciplinary context supported by cardiologists, physiotherapists, nurses, occupational therapists, dietitians, pharmacists, psychologists, and social workers.

In Belgium, patients receive reimbursement for involvement in CR (ie, maximum of 30 in-hospital sessions, 45 outpatient sessions), provided the patient meets certain criteria [13]. In Ireland, free standard CR services are generally offered (without reimbursement) to individuals. Unfortunately, particularly in Ireland, some CR units have suffered cutbacks in recent years.

The purpose of this study was to explore opinions and preferences to optimize PATHway intervention development before piloting the intervention (Trial registration number NCT02717806). This includes exploring the most appropriate content and viable approaches for PATHway intervention delivery. This was achieved through a comprehensive needs identification and analysis with PATHway patients and stakeholders using the capability, opportunity, motivation, and behavior (COM-B) model framework in two health care systems: Ireland and Belgium.

## Methods

### Interview Script Development

In-depth, semistructured, individual interviews were conducted in two trial locations (Dublin, Ireland and Leuven, Belgium). Interview scripts for both patient and stakeholders (see Multimedia Appendices 1 and 2) were developed using the COM-B model of health behavior change [10]. By adopting the COM-B framework, different aspects of behavior were investigated to design and implement the PATHway intervention appropriately. Author DW conducted all interviews in Ireland, whereas authors NC, VC, and RB conducted interviews in Belgium. All Irish interviews were transcribed by a selected transcription service, whereas in Belgium, all interviews were transcribed by the PATHway research team.

### Patient Recruitment

To target individual and interpersonal levels, 33 CVD patients were interviewed in a combined total across both sites (Dublin, Ireland n=20; Leuven, Belgium n=13). These two sites were selected as they were the locations of the PATHway study team clinical partners, and importantly, these sites were the locations of the phase 2 and phase 3 CR programs with access to patient and health professional cohorts. These specific locations facilitated an in-depth view of the varied CR practice across different European countries. No other sites participated in this discrete phase of development with patients.

Patients were recruited by the PATHway study team in conjunction with hospital and community partners. Participants from phase 2 (hospital-based) and phase 3 (community-based) CR programs with different levels of engagement in CR were approached. That is, four groups were identified: (1) those attending phase 2 (hospital-based CR), (2) those who enrolled but did not complete phase 2, (3) those who were engaged with phase 3 (community-based CR), and (4) those who dropped out of phase 3. For patients still engaged with CR programs in the hospital or in the community, announcements and information sheets were made available at the beginning and end of CR classes to explain the study, and contact details were given. However, for patients who were no longer attending CR programs, CR staff contacted them, and if interested in participating, potential participants were given contact details of the PATHway study team. Before participating in an interview, all patients were asked to complete the technology usage questionnaire [14] to obtain further information on the general and technological background of the patients. This questionnaire and related findings were published by the PATHway study team (see [14]). Patient recruitment and interviews conducted were determined by whether new information or concepts emerged from the interviews [15].

### Stakeholder Recruitment

The SEM was used to ensure inclusion of the full health ecosystem [11]. All stakeholders were approached by the PATHway study team following a brainstorming session with CR coordinators about potential participants to approach at each level of the SEM and each profession. All potential participants were then approached and given information sheets regarding the study and invited to participate. As such, interviews were conducted with a total of 21 stakeholders from each of the public policy, the community, and the organizational levels in Dublin (Ireland) and Leuven (Belgium): representatives from public policy, specifically individuals from the Department of Health (n=2) and from the Health Services Executive (n=1); representatives from the community, specifically general practitioners who refer patients to CR (n=3); public health nurses (n=1); local patient organization (n=1); national patient organization (n=1); representatives from the hospital, specifically the CR cardiologists (n=2); hypertension specialist (n=1); specialized cardiology nurses (n=3); physiotherapists or exercise physiologists involved in CR phase 2 and 3 (n=4); psychologist involved in CR (n=1); and technologists with experience of health care devices in CR (n=1). This sample endeavored to reflect the various levels and multidisciplinary nature of CR services insofar as possible at different specified levels of the SEM. This study aimed to capture the breadth of experience of multiple stakeholders across the CVD journey; however, it is acknowledged that potentially sufficient depth of these experiences may not be fully captured within this sample. Further sampling of each of the allied health professionals would be beneficial for future research as it was not possible within the scope of the current project to recruit further participants.

### Patient and Stakeholder Interview Analysis

All interviews were transcribed verbatim by the PATHway team. Data were subject to a thematic analysis, guided by Braun and Clarke’s [16] five-step framework and mapped to the COM-B framework. The five-step framework is listed as follows:

1. Step one includes familiarizing yourself with data through multiple readings.
2. Step two generates an initial list of ideas about what is in the data and what is interesting about them and involves the production of initial codes from the data.
3. Step three, themes begin to emerge, and this refocuses the analysis at the broader level of themes.
4. Step four involves reviewing themes whereby a set of candidate themes are explored and refined, including similarities and differences between interviews. This is an important step given the multisite approach in PATHway, which may offer conflicting findings.
5. Step five involves defining and naming themes.

Audit trails were used throughout to ensure transparency from raw interview transcripts to themes to use case formulations in both trial sites. Each sited coded all site-specific data separately. Preliminary coding was shared at each step of analysis as listed above, and group discussions were held. Authors from the Belgian site translated all emerging codes, and numerous representative quotes were translated from Dutch to English for the full team to discuss. All data were then combined to facilitate data synthesis and integration of qualitative data from both sites. This synthesis process was done in English and findings corroborated by authors from the Belgian site to ensure that codes and representative quotes were integrated appropriately. This synthesis of data was especially important given the need to reflect both the similarities but also the differences between the two health systems represented within this data. This analysis was done inductively and then mapped across to the COM-B framework to allow transparent translation to PATHway intervention requirements. Analysis was done separately for patients and stakeholders.

## Results

### Results of Cardiovascular Disease Patient Interviews

A total of 33 patients took part in individual interviews across the two sites. Thirteen patients (39%, 13/33) were from Leuven and 60% (20/33) from Ireland (mean age=60 years; female=21%, 7/33) and various levels of education (eg, second level education or lower=36%, 12/33; third level education [including undergraduate and postgraduate programs]= 64%, 21/33). There were 55% (18/33) from phase 2 and 45% (15/33) from phase 3 with various reasons for attending CR listed. There were varying levels of technology use with 58% (19/33) reporting high, 24% (8/33) moderate, and 18% (6/33) low technology use. An overview of the patient sample is shown in Table 1.

Textbox 1 represents the main themes and subthemes that emerged from the individual patient interviews.

### Theme 1: Capability

#### Physical Capability

Capability was first explored with CVD patients to establish whether individuals felt they were “physically capable” of engaging with PA. Patients were unsure of their physical fitness levels to engage fully with a home-based program. This lack of confidence in their own physical ability highlights the need to introduce exercises at a suitable level and carefully monitor participant satisfaction following each session.

Some patients raised the issue of how they were concerned whether the exercises would be “age-appropriate,” including any potential negative impact on comorbidities that some patients felt was an issue in their capability of initiating and maintaining PATHway use:

That circuit in the gym, I do have difficulties when I run...My hip starts to ache.

Participant 11, male, 65 years, low technology use, Leuven

#### Psychological Capability

##### Psychological Readiness

Apprehensions were raised about potentially not having the “psychological capability” or knowledge of CVD to know what physiological reactions are acceptable to experience during remote exercise participation. Patients suggested that information should be provided on what to expect while exercising, especially in relation to any symptoms that may suggest an adverse event. This was suggested to help understand their “new normal.” One patient stated:

I didn’t really know anything about physical activity. Now thanks to this program, I learned that it takes a lot more than you would think at first.

Participant 10, male, 42 years, high technology use, Leuven

Patients were interested to understand the different readings from devices and knowing safe and optimal ranges for exercise:

I’ll be anxious maybe initially to make sure that I’d be doing it within the confines that I’m supposed to.

Participant 1, male, 62 years, moderate technology use, Dublin

Further educational training (ie, information on optimal target heart rate zones, recommended daily step counts, and recommended weekly minutes of PA) was suggested as something to enhance capability so that patients could understand different physical measurements and what they mean for their CVD risk profile. Interestingly, although many patients had attended and engaged with both phase 2 and phase 3, many felt a lack of knowledge and capability surrounding the type of exercises to be conducted at home. Exercise examples and information regarding specific exercises, muscle groups, training types, and their respective benefits were listed as areas of interest that PATHway could provide. One patient stated:

I’m not sure, we’re piecing it together what we do myself and my wife. I wouldn’t be totally confident now that it’s a perfect training programme.

Participant 26, male, 60 years, high technology use, Dublin

##### Technology Readiness

Although capability for using technology to self-manage CVD among the target population was quite high, users were less confident in their ability to “set up” the PATHway system for the first time. This suggests that there may be an important role of mentoring within PATHway. One patient stated the following:

I wouldn’t have a clue but my son or daughter would. I wouldn’t have a clue how to set it up.

Participant 17, female, 36 years, high technology use, Dublin

Familiarization with the system was seen as crucial as patients wanted a face-to-face demo with the PATHway team initially. Information technology support and phone support were suggested as the most acceptable, with further suggestions of email support and YouTube videos for technically capable users. Some patients suggested a manual with diagrams and pictures with a quick guide to enable ease of use:

I think once you’ve had one session with people and they show you how to use it, it would probably be ok and maybe then if there was a place that you could go...if you weren’t able to manage it.

Participant 14, female, 74 years, moderate technology use, Dublin

### Theme 2: Opportunity

#### Physical Opportunity

Overall, patients expressed three common obstacles to physical opportunity. This was with particular reference to resources such as time, equipment (including high speed Wi-Fi), and space in the home. Patients showed various preferences in relation to having a set space for PATHway use at home vs a moveable system to facilitate varying family and home life needs. Flexibility of class start times within PATHway was seen as a positive feature for those who were still working; however, many felt that with flexibility came the trade-off of procrastination and no set time therefore leading to less accountability:

We all have the best intentions. So I think to have facilities at home...you want to be motivated, unless there was something you had signed up to.

Participant 16, male, 67 years, high technology use, Dublin

**Table 1:** Demographics of patients in qualitative interviews (Leuven and Dublin sites).

ID	Site	Phase	Technology use	Sex	Age (years)	Education	Reason for attending CR^a^
P1	Leuven	Adult congenital heart disease (phase 3)	Moderate	Male	25	PhD-student	Congenital heart disease
P2	Leuven	Phase 3	Moderate	Male	63	Master	Percutaneous coronary intervention (PCI)
P3	Leuven	Phase 2	High	Male	59	Master	PCI
P4	Leuven	Phase 2	Moderate	Male	50	Bachelor	Coronary artery bypass graft (CABG)
P5	Leuven	Phase 2	Low	Male	69	Master	PCI
P6	Leuven	Phase 3	Low	Male	64	Bachelor	PCI
P7	Leuven	Phase 3	High	Male	78	PhD (professor emeritus)	CABG
P8	Leuven	Phase 3	Moderate	Male	74	Master	CABG, ICD
P9	Leuven	Phase 2	Low	Male	48	Leaving certificate	CABG
P10	Leuven	Phase 2	High	Male	42	Bachelor	Heart failure
P11	Leuven	Phase 2	Low	Male	65	PhD	Heart failure
P12	Leuven	Phase 2 (dropout)	High	Male	55	Leaving certificate	CABG
P13	Leuven	Phase 3	High	Male	69	Bachelor	PCI
P14	Dublin	Phase 3 (dropout)	Moderate	Female	74	Bachelor	Heart attack or stenting
P15	Dublin	Phase 2	Moderate	Male	62	Intermediate or junior or group certificate	Heart attack or stenting
P16	Dublin	Phase 2	High	Male	67	Leaving certificate	Heart attack or stenting
P17	Dublin	Phase 2	High	Female	36	Intermediate or junior or group certificate	Heart attack or stenting
P18	Dublin	Phase 2 (dropout)	High	Male	61	Intermediate or junior or group certificate	Stenting
P19	Dublin	Phase 2	High	Male	70	Primary level	Heart attack or stenting
P20	Dublin	Phase 2 (dropout)	High	Male	60	Diploma or certificate	Stenting or pacemaker implantable cardioverter d*efibrillator*
P21	Dublin	Phase 2 (dropout)	High	Female	56	Intermediate or junior or group certificate	Heart attack or stenting
P22	Dublin	Phase 2	Moderate	Male	60	Bachelor	Heart attack or stenting
P23	Dublin	Phase 2	High	Female	46	Diploma or certificate	Heart attack or stenting
P24	Dublin	Phase 2 (dropout)	High	Male	49	Leaving certificate	Heart attack or stenting
P25	Dublin	Phase 3 (dropout)	High	Male	53	Intermediate or junior or group certificate	Prediabetes
P26	Dublin	Phase 3(dropout)	High	Male	60	Postgraduate or higher degree	Heart attack or stenting
P27	Dublin	Phase 2	High	Male	62	Leaving certificate	Bypass grafting
P28	Dublin	Phase 3	Low	Female	82	Diploma or certificate	Stenting
P29	Dublin	Phase 3	Moderate	Male	72	Master	Heart attack or stenting
P30	Dublin	Phase 3	High	Male	62	Leaving certificate	Heart attack or stenting
P31	Dublin	Phase 3	High	Female	—	Bachelor	—
P32	Dublin	Pilot phase 3	High	Male	62	Bachelor	Valve surgery
P33	Dublin	Phase 3	Low	Female	77	Diploma or certificate	Stenting

^a^CR: cardiac rehabilitation.

**Patient themes and subthemes.**

**Capability**

- physical capability
- psychological capability, including psychological readiness and technological readiness

**Opportunity**

- physical opportunity
- social opportunity

**Motivation**

- goal setting
- social interaction
- perceptions
- structured approach to exercise
- personalization
- present and future health and well-being

#### Social Opportunity

Patients felt a high level of support from their social environments, including family and friend contexts. However, a number of patients felt that exercise had to be self-motivated to ensure long-term maintenance. Several patients suggested that PATHway could be introduced as a family-wide intervention. Leveraging family connections to promote intergenerational lifestyle could enable PA, while not necessarily prioritizing it above other family commitments, as illustrated in the following statement:

I don’t want to become obsessed and say “no kids I’m not talking to you tonight, forget about the homework I’ve got my run.” I did that for long enough you know and now I’m trying to match everything up, not to be too greedy with my own time.

Participant 26, male, 60 years, high technology use, Dublin

Patients felt that they would like a bridge between hospital-based CR and community-based CR to maintain social connections forged within the hospital-based CR program:

We’re with people we all know, we know how each other feel. We’re with each other three times a week...If we could stay together, we continue together.

Participant 17, female, 36 years, high technology use, Dublin

This included connections with health care professionals (HCPs). PATHway was seen as a potential way of on-going professional support; however, an agreed structured approach to monitoring was seen as desirable:

Would I use it? YES! At least, if I’m still allowed to come on a consultation from time to time.

Participant 9, male, 48 years, low technology use, Leuven

Home exercise was seen as a positive alternative to gym use for long term maintenance. Patients shared concerns regarding dislike of gyms, embarrassment, and feeling out of place in the gym context:

You go to a gym, you’re looking at the person next to you, you’re wondering are they looking at you. For somebody who is overweight they don’t want to go to a gym and have anybody look at them. They feel insecure. If you’re at home you don’t have that.

Participant 23, female, 46 years, high technology use, Dublin

### Theme 3: Motivation for Cardiovascular Disease Patients

Various factors were explored in relation to motivation. Central subthemes were as follows: (1) goal setting, (2) social interaction, (3) perceptions, (4) structured approach to exercise, (5) monitoring, (6) personalization, and (7) present and future health and well-being.

#### Goal Setting

Goal setting was a key motivator for most of the patients; most identified that they felt less obligated to exercise at home. Patients felt that tracking PA and creating concrete action plans were key to success. Some patients wanted to set these goals with HCPs or face-to-face to create a sense of accountability, such as a contract agreement. Patients highlighted the need for personal fitness goals and functional improvement:

I’d be checking to make sure I’d be getting near my goal and if I wasn’t getting near then I’d make the extra effort to do it.

Participant 21, female, 56 years, high technology use, Dublin

Patients wanted progress and indicators of performance to be tracked to enable visual feedback, as well as physical testing at intermittent periods. Risk profiles were deemed important information by patients as many expressed not fully understanding the underlying cause of their CVD. Prompts were also seen as an important and acceptable part of engaging in maintenance of lifestyle change.

#### Social Interaction

Social interaction was the motivating factor for individuals currently partaking in CR and general PA. Many patients were interested in PATHway, allowing them access to a further social network or an avenue to augment their existing network:

You meet the other people who are in the same boat you’ll all have similar stories. So that alone means that you’re not alone.

Participant 32, male, 62 years, high technology use, Dublin

I like to talk, I like to have people around me and I have always been together with people. Being on my own is nothing for me.

Participant 3, male, 59 years, high technology use, Leuven

The group was also cited as creating a positive social pressure; however, some patients were concerned about unwise comparisons with fellow patients:

There’s no sense in comparing me with a 20 stone man. It’s inaccurate you know.

Participant 15, male, 62 years, moderate technology use, Dublin

#### Illness Perceptions

Participant’s personal illness perceptions (ie, their current health or CVD status and its subsequent impact) largely influenced participant views of their personal exercise ability and subsequent participation. Many patients did not consider CVD to be a chronic condition that needed to be managed and had difficulty understanding their personal risk profiles, with a majority of patients assigning their CVD risk factor to family history.

Certain exercises and sports were associated with specific age groups. Many patients were not active before the CVD incident and felt that fitness was purely functional, with many experiencing low self-efficacy in relation to exercise. This had an interesting impact on how different potential components of PATHway were received by participants. The gaming module of PATHway was often not considered as a viable option for rigorous targeted exercise as it did not fit with many of the participant’s traditional perception of what exercise “should be.” Some felt that implementing a “game” as a health care self-management solution was not appropriate:

It wasn’t used as an exercise thing; it was just something to do.

Participant 19, male, 70 years, high technology use, Dublin

Conversely, many users were interested in a low-technology solution such as engaging with PATHway through the Active Lifestyle component. Key motivators were enjoyment of the outdoors, engaging in exercise with a purpose, and getting out of the house.

#### Structured Approach to Exercise

A key point that certain individuals felt was necessary was a structured option to create a sense of obligation to engage:

Rather than going off and doing something which would not be of any benefit for me but would nearly be detrimental I think having a kind of a laid down programme, you’d use different bits of it but it’s the programme that suits your physical state, would be a very good idea.

Participant 32, male, 62 years, high technology use, Dublin

#### Monitoring

Monitoring, feedback, and HCP’s recommendation were key aspects of motivation to engage in PA. Patients’ felt that feedback could be delivered in a variety of ways including motivational messages, recordings, videos, immediate audio and visual feedback from the avatar and wrist-worn sensors, summaries, and graphs. However, patients noted that they did not want to become obsessive with the monitoring:

I think I’d become obsessed. I’d like to be able to go and say check my own when I need to but not to be in my face all the time.

Participant 23, female, 46 years, high technology use, Dublin

Patients valued health information and feedback could be utilized by an HCP but wanted their information to be private. Interestingly, patients referred to the PATHway system and remote monitoring as a “big brother” type of device:

It’s a bit like big brother as well...I personally think it would work.

Participant 21, female, 56 years, high technology use, Dublin

#### Personalization

Many patients felt that this individualization and monitoring would give them a greater insight into their personal health. Personal data was seen as a way to further their own awareness and self-reflection and aid the management of CVD. A further aspect for patients was the ability to control the exercise intensity, as illustrated in the following statement:

So long as it’s encouraging and it recognises people not as a number as such, as an individual...that to me now would be a big; I feel everybody kind of is inclined to be a number nowadays. That would motivate me as well.

Participant 23, female, 46 years, high technology use, Dublin

#### Present and Future Health and Well-Being

Prevention of further CVD incidences was a core motivator for many patients. Patients noted that there was an immense sense of achievement following exercise:

I know people who have had similar problems to me and they just haven’t bothered but for me it was the right thing. I still think it’s a great idea; it’s one way of making sure that I’m staying fitter and I’m doing the right things for the heart muscle to keep me going.

Participant 32, male, 62 years, high technology use, Dublin

PA was seen as a core component of stress management by patients who exercised regularly:

If you’re really focused on something...all the normal day to day stresses and everything are gone...and I like that.

Participant 31, Female, age not given, high technology use, Dublin

### Results of Stakeholder Interviews

Stakeholder interviews provided information from the stakeholders’ perspective of the key barriers preventing and the motivators increasing the likelihood of stakeholders in general to use PATHway as a tool to facilitate long-term maintenance of health behavior change among community-based CR patients. Stakeholders also reflected on elements that would act as barriers and motivators to engage. Stakeholders reflected on both professional and patient elements within this model.

Stakeholders considered aspects of patient engagement within community-based CR programs (eg, the psychological capability of some of the cohort to engage with new technology). However, there is also professional reflection in terms of barriers (eg, whole team buy-in). These themes are presented in Textbox 2 using the COM-B model framework.

### Theme 1: Capability

Capability was discussed in light of both psychological and physical capability for both patients and stakeholders.

#### Psychological Capability

##### Mismatch Between Current Cardiovascular Disease Patients and Future Cardiovascular Disease Patients

Many stakeholders suggested that future CVD patients in the coming years will become increasingly knowledgeable of health care technology and its use in self-management; however, some were unsure whether current typical profile CVD patients would be familiar enough with technology and willing to use it as part of their long-term CR:

There’s going to be new generation of patients going into the future that are more tech savvy than my generation.

Stakeholder 14, specialized cardiac nurse, Dublin

Other stakeholders recommended that data protection was an important issue, and all information recorded and retained by the system would need to be explained in full to the patient with details of why such information is necessary or beneficial:

This is like Big Brother...they would need to be reassured…it is people watching what’s going on but for a very good reason...there’s an extra element there with people who haven’t grown up with this as part of normal experience.

Stakeholder 15, health service executive, Dublin

**Stakeholder themes mapped to capability, opportunity, motivation, and behavior (COM-B) model.**

**Capability**

- psychological capability
  - mismatch between current cardiovascular disease (CVD) patients and future CVD patients
  - ease of use
- physical capability
  - alarm and emergency protocols

**Opportunity**

- social opportunity
  - peer-to peer social connections
  - health care connections
  - whole team buy-in
  - general social support
  - patient-led participation
- physical opportunity
  - finite resources

**Motivation**

- potential for real-time assessment and feedback
- technology augmented care
- positive patient reinforcement
- personalization
- feedback

##### Ease of Use

Stakeholders emphasized that the system above all else needed to be easy-to-use for both clinicians and patients. Overly complicated systems were seen as a barrier to use. Stakeholders also suggested that the avatar should be able to issue teaching points as well as visual cues to the patient to aid interaction with the system, including an explanation of how to wear and use the sensors within PATHway. One stakeholder stated:

We’re dealing with patients putting on monitors every day and it can be very difficult for some of them, technically very difficult.

Stakeholder 14, specialized cardiac nurse, Dublin

Stakeholders felt that information technology support should be made available for patients:

I think people don’t mind being talked through something over the phone but certainly if you’ve to go online and try and chat to someone I think there’s a barrier.

Stakeholder 13, local patient organization, Dublin

This ease of use theme also included a clinical and patient interface. Stakeholders suggested that summary information would have to be presented in a simple easy-to-use way, including graphics of patient progress and the information and progress in relation to important risk factors. These summaries were recommended to be no longer than 1 page long and easily read during a consultation with information that did not just provide a duplicate of what is already available:

[People are] terribly impatient with stuff, you know? It has to be the cat sat on the mat

Stakeholder 1, cardiac rehabilitation cardiologist, Dublin

#### Physical Capability

##### Alarm and Emergency Protocol

Stakeholder emphasized how clearly the system needed to alert the patients to adverse events. Stakeholders also highlighted the need to explain the potential for false alarms to patients before interaction with the system:

You need a big red stop...Stop what you’re doing, sit down, have a glass of water, in big writing and it has to be red, because that’s alarm.

Stakeholder 14, specialized cardiac nurse, Dublin

### Theme 2: Opportunity

In terms of opportunity, stakeholders reflected on aspects of both social and physical opportunity. This included how patients engage but also how HCPs may also engage (see subthemes “whole team buy-in” and “patient-led participation”).

#### Social Opportunity

##### New Social Peer-to-Peer and Health Care Connections

To ensure patient engagement with PATHway, stakeholders suggested that PATHway should aim to harness initial hospital-based social connections for optimal results in facilitating recruitment and retention among current CVD patients within the hospital. By using hospital-based CR as a platform to launch and familiarize PATHway with patients, preexisting groups and social interactions can be augmented, whereby patients have the option to motivate one another, provide peer mentorship, and strengthen habit-forming and routine created during attendance at the hospital-based CR. One stakeholder stated:

There's also the sense of belonging to a group, which is very attractive for some patients.

Stakeholder 2, public policy, Dublin

Stakeholders felt that PATHway could be seen as a type of remote follow-up for patients to provide additional support and as a way to further educate and inform patients of best practice and current guidelines. This was deemed, by the stakeholders, to be integral to on-going patient self-management:

There is that instant ready assessment of how they’re doing with a straight access to whoever the key players are including the patient and their health professional.

Stakeholder 13, local patient organization, Dublin

A “patient-centered approach” was deemed very important, particularly in terms of engagement and long-term use of the system:

It is not only about what you think is important but also what the patient thinks is important, it always the combination.

Stakeholder 1, cardiac rehabilitation cardiologist, Dublin

##### General Social Support

Some stakeholders suggested the participation of family and peers is a practical way to provide implement a “patient-centered approach.” Peer mentors who have experienced CVD are in a unique position to guide, advise, and motivate fellow CVD recoverees to engage with CR and PATHway in particular. Family members can be active within the intervention as support in PA maintenance, potentially playing a monitoring role to create accountability for the CVD patient, as illustrated in the following statement:

The participation of other members of the family, I think that’s really positive and I think children, younger people love to take on and support a parent particularly in getting better you know. So I think there’s huge potential there...we know it from other settings, the peer and the group initiative.

Stakeholder 13, local patient organization, Dublin

##### Whole Team “Buy-In”

“Buy-in” from the cardiology unit was seen as crucial to the implementation of such an intervention, including consultants and senior staff members. The “buy-in” of the HCP was seen as an advantage with regards to patient recruitment and retention, and whole team “buy-in” was ideally envisaged as a multidisciplinary effort to optimize patient experience.

Stakeholders within the Belgian system felt that further information on past patients would be valuable and rewarding, whereas within the Irish system, this extra information and follow-up seemed to create a sense of further responsibility, as illustrated in the following statements:

I would feel satisfied to objectively see that the patients keep exercising. I consider it very important and I've been missing this aspect throughout my years at the cardiac rehab. What is the long-term results of all our investments, ie, time and effort we put in the patients to clarify to him what is important the long term.

Stakeholder 2, public policy, Dublin

That’s a whole new role though...I wouldn’t feel comfortable doing that in my daily work because I have my own work to do and that’s a responsible job you know? You’ve people’s lives at risk at home...they put a lot of onus on their own health, their responsibility on to your shoulders and that can be difficult.

Stakeholder 14, specialized cardiac nurse, Dublin

##### Patient-Led Participation

Several stakeholders identified that engagement with PATHway needs to be patient-led. Stakeholders identified that if a patient requests information from the PATHway system to be a part of their consultation then it “forces” them—the stakeholders—into action and engagement with the system and can generate a good foundation for further focused follow-up appointments, as illustrated in the following quote:

Basically, they’re patient held, they’re patient produced and it’s part of the consultation. If you’re going to start to ask primary health care to provide it at another time access a portal of some sort to view patient data I think there’s challenges around the uptake of that.

Stakeholder 21, GP, Dublin

#### Physical Opportunity

Stakeholders discussed barriers to PATHway implementation that were mainly focused around resource issues.

##### Finite Resources

Time and money were seen as finite resources within the clinical setting. A key concern regarding implementation of PATHway within a clinical setting is whether PATHway would deliver better patient care and useful clinical information while maintaining or minimizing current workload:

These are serious time invests...If you really want to do an intensive follow up it surely would be a serious time-investment compared to just receiving a weekly summary where you can see how frequent and at what intensities the patient has been training.

Stakeholder 2, public policy, Dublin

Further concerns were raised regarding the follow-up of patients and whether this task fit into existing CR roles and whether the obtained information would be used in existing practices. This highlighted the need for an examination of intended financial and staffing resources:

We’re giving them information to go on to do [community-based CR] but we don’t bring them back.

Stakeholder 14, specialized cardiac nurse, Dublin

### Theme 3: Motivation

#### Age of Measurement

Stakeholders identified that technology as an integral part of future health care, calling it the “age of measurement” where patients and HCPs alike expect to have health status measurements and feedback. Giving feedback and providing various health measurements was identified as increasing the acceptability and adding value to the use of PATHway among patients:

I think it can or could offer added value for certain topics, especially in terms of objectivizing a number of parameters and general items that we are often still evaluating in an approximate or very subjective way.

Stakeholder 2, public policy, Dublin

HCPs felt that these measurements could increase efficacy within patient consultations and act a good starting point for follow-up appointments.

#### Mode of Feedback

Feedback was suggested in a variety of forms particularly in relation to information available through PATHway and also push notifications and prompts:

Technology can sometimes be a reminder, a stimulus in order to motivate your patients to practice, so that they can rehabilitate and make some progress.

Stakeholder 2, public policy, Dublin

It was advised that patients should give feedback to aid tailoring and program improvement. This was suggested to be easily captured via brief feedback from the participant on their satisfaction and enjoyment levels following their exercise class.

Many stakeholders mentioned the issue of patients becoming too dependent and obsessed with measurement and numbers (ie, with wrist worn sensors, etc). This was a cause for concern, as ideally patients should feel safe to exercise limits using the Rate of Perceived Exertion scale, and this would be a better gauge of progress, as illustrated in the following statement:

They become obsessed with the numbers and if there’s any fluctuation they start panicking so that’s why I don’t think it’s, personally, it’s a good method. And we have always used the RPE scale here and I find it works very well if you explain it properly to the patient.

Stakeholder 14, specialized cardiac nurse, Dublin

In terms of feedback for stakeholders, key outcomes of interest were as follows: body mass index, height, weight, cholesterol, alcohol consumption, smoking cessation, medication adherence, blood pressure, steps, PA (Frequency, Intensity, Type, and Time), nutrition, well-being, general mental health, quality of life, satisfaction using PATHway, self-efficacy, engagement with pathway, social support, and social interaction.

#### Technology Augmented Care

Some stakeholders expressed that patients should be educated in a holistic way, including exercise confidence and psychological well-being. This was seen as important outcome to facilitate a positive view of health and self-management, potentially having an impact on illness and exercise perceptions:

I think it is also important to validate the psychological well-being of the patients...does the patient feel better, are patients more confident while exercising and is he more motivated to exercise.

Stakeholder 12, psychologist involved in CR, Dublin

Stakeholders clearly identified that although technology could augment care and many were very supportive of the role of technology in health care whereby a system such as PATHway would be able to complement existing care practices and lead a more efficient and satisfactory follow-on service:

It would make it possible to interact with the patients on a regular basis besides the more formal contacts we have during the follow-up consults.

Stakeholder 6, exercise physiologist, Leuven

However, several stakeholders clearly raised the issue that the therapeutic relationship should not be replaced totally:

You...would be very wary of programmes...taking over...the irreplaceable role of the therapeutic relationship...I only think of it as augmentative.

Stakeholder 12, psychologist involved in CR

Technology was seen something that could provide comfort and safety to CVD patients who lacked confidence while exercising:

The greatest challenge for me is to translate the feeling of safety to the patient, that they really get the feeling “I can exercise here safely.” I think that’s the most important.

Stakeholder 10, exercise physiologist, Leuven

#### Positive Patient Reinforcement

Positive reinforcement in the absence of negative reinforcement was seen as important in terms of motivation and engagement. Many stakeholders suggested a multidisciplinary approach whereby patients set goals in tandem with key health professionals:

You should do it in cooperation with all healthcare providers involved and set up certain goals together with your patients on beforehand.

Stakeholder 4, exercise physiologist, Leuven

Positive reinforcement was a key point that was mentioned in terms of feedback to the user. Nonconfrontational gradual approaches were seen as a productive way to move forward with health and lifestyle information and intervention in a primary care setting:

Setting goals together with the patients and evaluating with which means you'll try to achieve those goals. If you impose a certain technology to your patients, in whom they don't fully believe themselves, you'll fail. It must be integrated within some kind of motivational strategy.

Stakeholder 4, exercise physiologist, Leuven

#### Personalization

Personalization was cited to be key for both users and clinicians. Provision of personalized exercise programs was deemed important in terms of patient capability and enjoyment:

There has to be provided something whereby the patient himself could personalize his therapy and be personally responsible together with the health care provider and also helps shaping his own programme.

Stakeholder 4, exercise physiologist, Leuven

## Discussion

### Principal Findings

This study addresses both patient and stakeholder requirements for a CVD eHealth intervention. This development work highlights the need for certain aspects of capability, opportunity, and motivation to be addressed for the implementation of an eHealth self-management system among CVD patients and stakeholders. This study highlights the core combined requirements of such a system for both patients and other key stakeholders. In summary, Textbox 3 broadly highlights high-level needs such as (1) personalized feedback delivered through easy-to-use technology, (2) facilitating social connection and support (both peer and professional), (3) the importance of the availability of resources, and (4) augmentation of existing relationships through technology.

Initially, before patients began PATHway use, physical and psychological capabilities were felt to be important considerations in terms of use of the system. Participants felt that the PATHway system should be as integrated into daily living as possible. Passive data collection or “sensed data,” including heart rate, steps, calories, and minutes of PA will allow a wide range of information to be available for the users to get up-to-date feedback facilitating engagement with behavioral change components of PATHway. It has been shown that personalized interventions have superior efficacy over time compared with those that base their tailoring on single or infrequent assessments (eg, baseline) [17,18]. This also creates a balance of user engagement but also allows flexibility around potential participant difficulties or reluctance to enter information manually.

Once issues of capability had been discussed, it was apparent that patients valued information and measurement of their health status. However, despite the desire for improved measurement of core outcomes and increased reporting, stakeholder and patient enthusiasm for “the age of measurement” must be tempered with caution, as both patient and stakeholders had reservations in relation to patient technology competencies. This concern was reflected in the patient theme “psychological capability” and subthemes “psychological readiness” and “technological readiness,” whereas stakeholders flagged a “mismatch between current CVD patients and future CVD patients” technology competencies. Digital health literacy is an important consideration within eHealth interventions, and participants need to be supported appropriately within intervention standard operating procedures [19]. Potential solutions for PATHway include a user manual, a familiarization phase within CR classes, how-to videos, and a support line. These options will be further explored within the PATHway trial and further qualitative work in PATHway debrief interviews. These will be guided by the Health information technology Usability Evaluation Model [20] and will be used to evaluate the experience with selected SEM stakeholders.

Despite these concerns regarding digital health literacy, findings from previous studies [14] suggest that the current target CVD population *are* familiar with technology and, importantly, have regular access to smartphones and the Internet, with the majority of patients reporting the concept of PATHway as appealing. These findings support previous formative work from a mobile health (mHealth) CR exercise intervention in New Zealand [21]. However, it is important to embed this formative work within the context of recent evidence synthesis highlighting key behavior change techniques to be used in eHealth interventions with CVD patients [22,23]. eHealth offers a tangible opportunity to provide pervasive connections between CVD patients and HCPs, thus “harnessing phase 2 connections” (as recommended by key stakeholders) for optimal phase 3 CR adherence and engagement.

Findings revealed that “the instructor” was an essential component of the theme “motivation” for patient CR adherence. Previous research has cited the use of an avatar within mHealth interventions as a potentially “nonthreatening conversational agent” [24]. Previous research has shown that often patients perceive avatars positively, assigning high levels of empathy and alliance to the avatar [25]. Significantly, this also has implications for patients with low levels of health literacy. From the qualitative interviews, it was clear that many patients did not engage with the health information and guidance provided for them by traditional HCPs and stakeholders in phase 2.

**Combined patient and stakeholder capability, opportunity, motivation, and behavior (COM-B). Italics indicate COM-B subcategories.**

**Capability**

- psychological capability
  - mismatch between current cardiovascular disease (CVD) patients and future CVD patients
  - ease of use
  - psychological readiness
  - technological readiness
- physical capability
  - alarm and emergency protocols

**Opportunity**

- social opportunity
  - new social peer-to-peer and health care connections
  - whole team buy-in
  - general social support
- Physical opportunity
  - finite resources
  - clinical and patient interface

**Motivation**

- patient-led participation
- mode of feedback
- potential for real-time assessment and feedback
- positive patient reinforcement
- personalization
- goal setting
- social interaction
- perceptions
- structured approach to exercise
- present and future health and well-being

Perhaps the use of an avatar or “conversational agent” within PATHway can initiate engagement with certain at-risk individuals who would otherwise not engage with regular CR routes of care.

The theme “opportunity” relates to both “physical opportunity” and “social opportunity.” Within the subtheme “social opportunity,” a “community-of-practice” was called for with the implementation of a family-wide intervention and workplace intervention to enable greater adoption of CR guidelines. This suggestion from patients and stakeholders to use the CVD incident as an opportunity or “intervention window” whereby patients and their families use the incident of CVD to promote healthy behavior change within their home. This finding is important for future CR interventions to optimize engagement initially within the intervention but also within eHealth trials for the purposes of recruitment and retention. This use of a “teachable moment” has been used in previous PA interventions to leverage a greater impact than stand-alone individual intervention components [26].

Finite resources were an issue for stakeholders. A key goal for eHealth interventions is to provide an effective, evidence-based, low-cost alternative to traditional health care routes. eHealth can reduce the burden of patient management through enabling remote participation in a phase 3 CR program. However, key stakeholders have identified issues with role delineation and remain concerned regarding the burden of remote patient monitoring in conjunction with their existing duties. This highlights the importance of the stakeholder identified theme “whole team buy-in” and consideration of the health ecosystem. This is particularly relevant given the different contexts in which the PATHway system may be implemented within. The Irish Association of Cardiac Rehabilitation has stated that CR services in Ireland have received cutbacks during a time of economic recession in Ireland. It is therefore noteworthy that Irish stakeholders found the prospect of further patient information and potential related duties and responsibilities difficult to envisage within their current context, whereas the Belgian stakeholders found these proposals predominantly positive and feasible to implement.

Goal setting and monitoring were integral elements of the patient theme “motivation.” Implementing goal setting is a key behavior change technique to be used within PATHway and one that was the most cited by patients as desirable. Previous research has examined implementing different levels of goal options to promote participant self-efficacy in relation to exercise [27]. This is very important for remote eHealth interventions given participant concerns regarding “physical capability” and “exercise perceptions.” Goal feedback is important to buffer against patient disengagement and integral to participant retention with the PATHway system. This relates back to the stakeholder recommendation of using mainly positive reinforcement. King and colleagues [27] employed positive reinforcement statements that were delivered when participants either met their weekly goal or exceeded their weekly goal. Future PATHway development phases will be able to explore how best to present such goal-setting and monitoring functions to foster motivation for participants. This finding is particularly relevant for studies that will implement exercise prescriptions for participants and adapt goals based on monitored progress.

Personalization was also expected in PATHway feedback, prompts, and notifications. This expectation is interesting as it has been previously demonstrated that tailored health messages are more engaging and effective in terms of health behavior change than untailored, generic messages [28]. As participant engagement and retention are critical factors in successful behavior change research, it is important for further exploration as considerable resources are needed to deliver tailored content.

Social interaction with others is a core driver of motivation and is closely linked to the “social opportunity” subtheme within the opportunity category of the COM-B model. Social opportunity can be defined as an opportunity created by cultural context that shapes how we think about things [10]. In essence, it can be seen that the social context (ie, social opportunity) facilitates initial engagement with an eHealth system such as PATHway (eg, your CR coordinator recommending its use). It has been previously evidenced that people are inclined to expose themselves to innovations that not only provide a solution to their needs but that also appear to be consistent with and reinforce their attitudes or value systems [29]. Further to this “social opportunity,” social interaction as a motivation focuses on the social connection that PATHway could potentially facilitate. This subtheme highlighted again the importance of the stakeholder call for harnessing phase 2 connections both in relation to the peer-to-peer social connections made but also the important health care connections. The importance of social interaction as a motivation for engagement with the PATHway system tied in with the stakeholder recommendation of a “patient-centered approach,” employing key strategies such as that of structured social support and peer mentoring for patients. Augmenting existing social connections can be effective in increasing PA, along with other healthy lifestyle behaviors. The harnessing of these existing connections may be more effective for patient engagement and satisfaction than trying to create a whole new online community.

### Strengths and Limitations

The use of qualitative interviews in this study allows in-depth understandings of PATHway patients and stakeholder views that may not be reached using quantitative methods alone, while the use of COM-B driven interview scripts facilitates evidence-based intervention development and highlights areas to address for future intervention implementation across different European health care systems.

The recruitment of a broad spectrum of users was an important task for PATHway and a key strength given the multisite nature of the project. Recruitment was balanced for age, gender, and socioeconomic status insofar as possible. However, in terms of limitations, “dropouts” from phase 2 and phase 3 were difficult to recruit because of their disengagement with services.

### Future Research and Conclusions

This is the first phase of development for the PATHway system. Further extensive user testing will be conducted, and a process evaluation in terms of the feasibility, acceptability, satisfaction, and usability of the PATHway system from the participants’ perspective will be assessed before a randomized trial. This future trial will also involve a health economics evaluation of the cost-effectiveness of PATHway. Furthermore, following this trial, debrief interviews will be conducted with participants and stakeholders (ie, as defined by the SEM used previously). This is crucial for future feasible implementation of the PATHway system within other European health care systems.

In conclusion, several broad learnings emerged from the in-depth qualitative work with individuals with CVD and HCPs. It is clear that a multifaceted, personalizable intervention is desirable. Key learnings include the need for maximal patient tailoring, simplicity within the platform, technology-augmented care, enabling or increasing individual self-management through eHealth, and capitalizing on an appropriate time to intervene in the CR journey.

## Appendix

Multimedia Appendix 1 Patient interview script.

Multimedia Appendix 2 Stakeholder interview script.

## Abbreviations

COM-B: capability, opportunity, motivation, and behavior
CR: cardiac rehabilitation
CVD: cardiovascular disease
eHealth: electronic health
HCP: health care professional
mHealth: mobile health
PA: physical activity
PATHway: Physical Activity Towards Health way
SEM: Social Ecological Model
