# Supplementary material for: Electronic Health Physical Activity Behavior Change Intervention to Self-Manage Cardiovascular Disease: Qualitative Exploration of Patient and Health Professional Requirements
Source: J Med Internet Res. 2018 May 8;20(5):e163. doi: 10.2196/jmir.9181 (PMC11340777; doi:10.2196/jmir.9181)
Supplement: Multimedia Appendix 1 [file jmir_v20i5e163_app1.pdf]

Patient Interview Script

Greeting and explanation		
• Hi my name is …….. and this is ………<br><br>• We are going to talk about physical activity, exercise and time spent sitting as we would like to get your thoughts on each of them.<br><br>• We are exploring the possibility of using technology within cardiac rehabilitation to help participants engage with CR long-term.<br><br>• You have completed a questionnaire for us, thank you. Now we would like to ask you a few more questions to find out a little bit more from your perspective.<br><br>• Please be honest when answering questions as there are no right or wrong answers.<br><br>• If it is ok, we will audio tape our discussion so we can go back and listen to it later. No one else will hear this tape and we will keep your name confidential.<br><br>**Start tape. Say the date, identification of interviewee participant number, recorder name (e.g., Monday, 16th February, The Mater Hospital, CVD patient XX, Catherine recording)**		
	Question	Prompts
Physical Activity		
Do you take part in physical activity?		
Capability	What do you think the difference between PA, exercise and time spent sitting is?<br><br>Do you feel physically able to take	e.g., even just your best guess

	part in physical activity?<br><br>Do you know how/ know what to do in order to engage in physical activity?	e.g., know exercises, skills etc.
Opportunity	Do you feel you have the resources to take part in physical activity?	e.g., time
Motivation	What motivates you to engage in physical activity?<br><br>What would make participating in physical activity more appealing to you?	e.g., why, how, could you tell me more about that?

Questions not classified but COM-B will be applied post interview
What kinds of physical activity do you take part in?
What kinds of exercise do you take part in?
What do you like about physical activity or exercise?
What prevents you from doing physical activity or exercise?

As we talk today, we will be referring to **physical activity** as any other activity that you do (e.g. going for a walk, gardening etc.), exercise is structured, planned activity (e.g. doing your CR exercises, being part of a class or a group-based exercise) and time spent sitting (or sedentary behaviour is when you do no physical activity or exercise.

Exercise Settings		
Do you exercise at home?<br><br>Do you exercise outside the home<br><br>(yes or no will dictate some of the following options)		
Capability	What exercise do you feel you can physically do at home?<br><br>What exercise do you feel you know how/have the skills to do at home?	
Opportunity	Do you feel you have the resources to exercise at home?	e.g., space, equipment
Motivation	Is there anything about exercising at home that is appealing to you?<br><br>OR<br><br>Is there anything about exercising at home that is not appealing to you?<br><br>Is there anything about exercising outside the home that is appealing to you?<br><br>OR<br><br>Is there anything about exercising	e.g., could you tell me more about that?

	outside the home that is not appealing to you?	
Questions not classified but COM-B will be applied post interview		
How did you find out about **identify specific CR programme **?		
What influenced you to join the programme?		
What will you do once it finishes? *(may be applicable to some only)*		
What did you like about it?		
Social Support		
Capability	n/a	
Opportunity	Are your friends physically active?<br><br>Are your family physically active?<br><br>Do you feel there is any encouragement/discouragement to be physically active from those around you?<br><br>How might others be more supportive of you participating in physical activity?	e.g., what do they do<br><br>e.g., Who? How? Could you tell me more about that?

Motivation	What support do you think would make you more likely to participate in physical activity?	e.g., could you tell me more about that
Technology Use		
When you think of technology for CVD patients to use to get physically active-what comes to mind?		
Capability	Do you feel you would have the skills to be able to use technology at home to be more physically active?<br><br>What are any issues you can think of that might stop you from being able to physically use the technology?	e.g., show demos-pictures, sensors, video clip.<br><br>e.g., being able to see the buttons, screen, buttons to small to press accurately etc.
Opportunity	Do you feel you have the resources to use technology at home to help you be more physically active?	e.g., time, space for a console, IT support
Motivation	Would you like a tech-based intervention to be part of your usual cardiac rehab care?<br><br>What does a tech based intervention need to have to be	e.g., why, how, could you tell me more about that?

	useful to you?<br><br>Would a personalised tech- based intervention be more or less appealing to you?<br><br>Do you think technology could help you be become more physically active?<br><br>How acceptable is it to you that you would incorporate technology into your daily life?<br><br>How likely is it that you will incorporate technology into your daily life?	e.g., Why? How? could you tell me more about that?<br>-personal details<br>-motivational messages<br>-sharing exercise performance with other participants<br><br>e.g., on a scale of 1-10, 1=not at all acceptable to 10= very acceptable.<br><br>e.g., 1= not at all likely to 10 = very likely.
Questions not classified but COM-B will be applied post interview		
What aspects of tech-based intervention might be challenging for you?		
