# Supplementary material for: Electronic Health Physical Activity Behavior Change Intervention to Self-Manage Cardiovascular Disease: Qualitative Exploration of Patient and Health Professional Requirements
Source: J Med Internet Res. 2018 May 8;20(5):e163. doi: 10.2196/jmir.9181 (PMC11340777; doi:10.2196/jmir.9181)
Supplement: Multimedia Appendix 2 [file jmir_v20i5e163_app2.pdf]

Stakeholder Interview Script		
• Hi my name is …….. and this is ………<br>• We are going to talk about physical activity, exercise and time spent sitting as we would like to get your thoughts on each of them.<br>• We are exploring the possibility of using technology within cardiac rehabilitation to help participants engage with CR long-term. We would like your views on this.<br>• Please be honest when answering questions as there are no right or wrong answers.<br>• If it is ok, we will audio tape our discussion so we can go back and listen to it later. No one else will hear this tape and we will keep your name confidential.<br>**Start tape. Say the date, participant ID, health professional role, recorder name, and facilitator name (e.g., Monday, 16th February, The Mater Hospital, Participant 1, CR nurse, Catherine recording, Michelle facilitating)**<br>□ What do you think is the difference between physical activity, exercise and time spent sitting?<br>□ As we talk today, we will be referring to **physical activity** as any other activity that you do (e.g. going for a walk, gardening etc.), exercise is structured, planned activity (e.g. doing your CR exercises, being part of a class or a group-based exercise) and time spent sitting (or sedentary behaviour is when you do no physical activity or exercise.<br>**Physical Activity**<br>**N.B Repeat definition of PA**		
Adopting Pathway as part of usual practice		
COM-B	Question	Prompt
What is your role within Cardiac rehabilitation? How do you perform this role?		

Do you expect that your role may improve due to the incorporation of ICT solutions?		
Capability	Do you feel you have the skills /knowledge to adopt technology use into your everyday work?	
Opportunity	Do you feel you have the resources at your disposal to implement technology use into your everyday work?<br><br>Do you feel your **organaisation** is a place that facilitates the incorporation of technology use into your everyday work?	
Motivation	Do you want to adopt technology use into your everyday work?	

	Do you feel you need to adopt technology use into your everyday work?<br><br>Do you believe that adopting technology use into your everyday work would be a good thing?<br><br>What plans could be put in place so that you could adopt technology use into your everyday work?	
Questions not classified but COM-B will be applied post interview		
On a scale of 1-10, how acceptable is it that you would incorporate technology into your daily life? (1 = not at all acceptable to 10 = very acceptable).<br><br>On a scale of 1-10, how likely is it that you will incorporate technology into your daily life? (1 = not at all likely to 10 = very likely).		
Clinical Requirements		

How might the evaluation of the performance of a CVD patient while he/she is exercising at home be useful?	
What kind of feedback would you expect, assuming that the system is able to extract a variety of information concerning the performed exercise?	
Which are the most important indicators denoting the successful adoption of the home care service?	
How do you evaluate patient adherence?	
Under which conditions would you (or would you not) undertake the responsibility to monitor a CVD patient during a cardiac rehabilitation program at home?	
Are phase 3 CR patients exercising at home generally efficient enough by themselves to reach good results	What could go wrong? Could a tech based intervention providing feedback overcome this?
What are the most important measurements/information you need to have in order to evaluate the progress/success of the exercise?	
As the home care intervention is by nature an unsupervised process, what	

information you think is sufficient in order to ensure day by day exercise safety?	
What does a tech based intervention need to have to be so that you would trust it, and your patients to use it in their own home?	What? Why? How would this work?
What type of remote feedback should a tech based intervention need to be able to provide you	What? Why? How would this work?<br>Would this improve patient management?
Besides exercise data, what other type of information from the patient would it be useful for you, in order to evaluate patient status and propose short term (day by day) and long term (monthly) adaptation to the CR program (e.g. daily vitals, life style information).	
Patient Requirements	
Do you think that the use of wearable sensors in such a system might cause dissatisfaction to the patient?	
How many sensors you think that could be used in order the patient to feel and stay comfort?	
Would a gamified tech based intervention cause stress in the patients by incorporating game elements such as	

time limits or score penalties?	
Would a tech-based solution incorporating video game elements (gamification) be an appropriate tool for increasing the motivation of phase 3 CR patients?	
What type of remote feedback would a tech based intervention need to be able to provide to the patient?	
What is a good practice in order to provide the patient with information regarding his/her physiological data/status?	(e.g.say “ presence of arrhythmia” or say “better to stop exercise for now”).
Tech Use Requirements	
From your perspective, when you think of technology for managing your CVD patients what comes to mind?	
When you think of technology for CVD patients what comes to mind?	
Do you think technology could help you CVD patients to become more physically active?	

What does a tech based intervention need to have to be useful to you?	
What aspects of a tech based intervention might be challenging for you?	• Content of programme<br>• Time management (possibly increased workload)<br>• Change of your routine in the clinic<br>• Trusting technology<br>• Safety<br>• Responsibility/accountability
How would you like this technology based intervention to be a part of your usual cardiac rehabilitation care?	
